# An introduction to biofilms: what they are, why they form and their impact on built and natural environments

**DOI:** 10.1099/mic.0.001338

**Published:** 2023-08-01

**Authors:** Natalie C. Bamford, Cait. E. MacPhee, Nicola R. Stanley-Wall

**Affiliations:** 1 Division of Molecular Microbiology, School of Life Sciences, University of Dundee, Dundee, DD1 5EH, UK; 2 National Biofilms Innovation Centre, School of Physics & Astronomy, University of Edinburgh, EH9 3FD Edinburgh, UK

## Abstract

Biofilms are complex communities of microbes that are bound by an extracellular macromolecular matrix produced by the residents. Biofilms are the predominant form of microbial life in the natural environment and although they are the leading cause of chronic infections, they are equally deeply connected to our ability to bioremediate waste and toxic materials. Here we highlight the emergent properties of biofilm communities and explore notable biofilms before concluding by providing examples of their major impact on our health and both natural and built environments.

## What is a biofilm?

The first observation of biofilms has been retrospectively credited to Antonie van Leeuwenhoek, a Dutch scientist in the 17th century, who used a simple homemade microscope to observe microbial communities in dental plaque and other environments. He noted that this material was composed of numerous microorganisms and described them as “animalcules”.

The term “biofilm” was introduced in the early 1970s by microbiologist J. William Costerton and colleagues, who were studying the bacterial communities that form on surfaces in aquatic environments. They found that these communities were not simply random aggregates of individual cells, but instead were highly organized structures with specialized adaptive functions, such as nutrient uptake, waste removal, and protection from environmental stressors.

**Figure 1:**
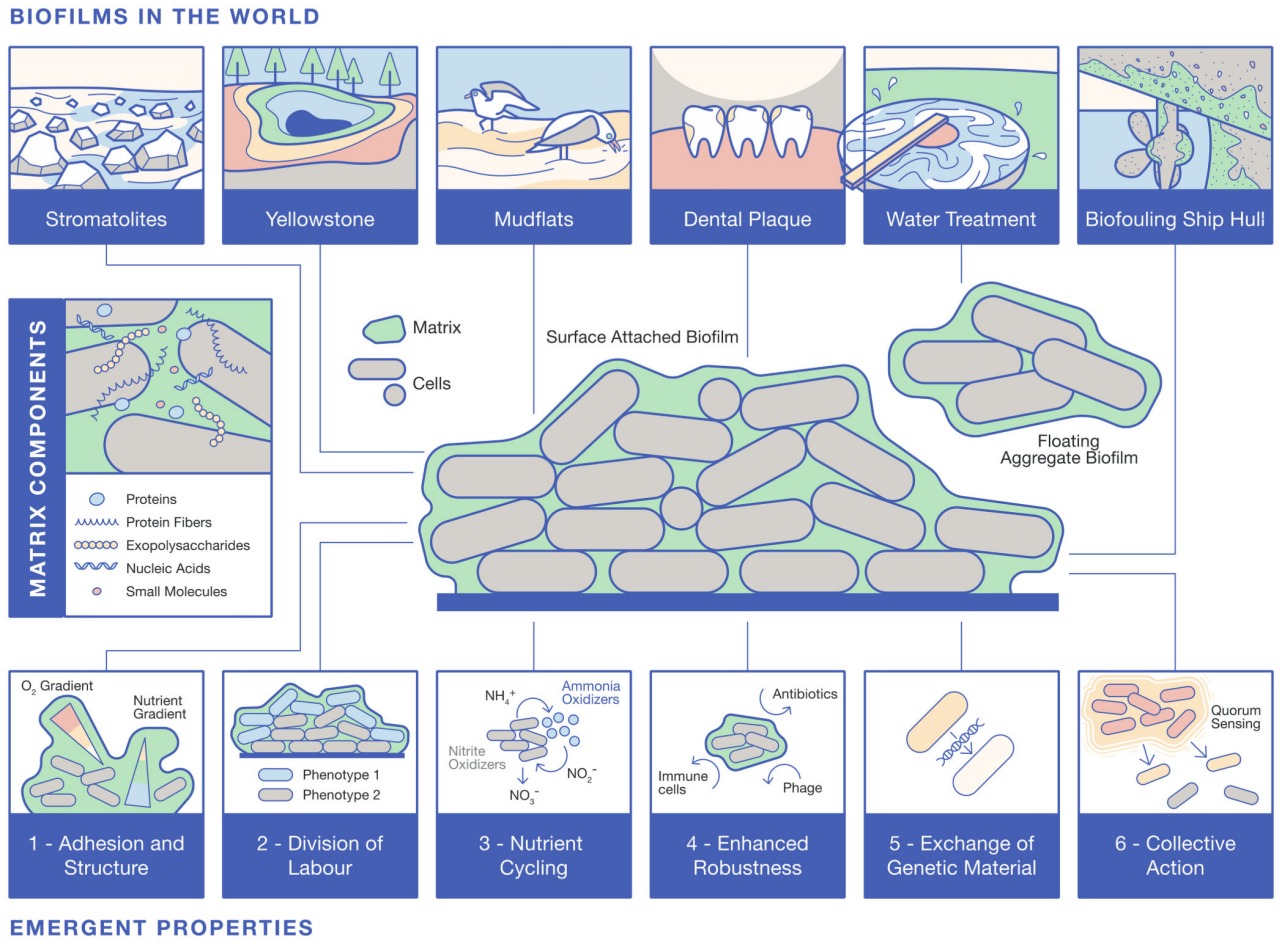
Biofilms across the scales of life: The central schematic shows the arrangement of mixed species of microbes embedded within an extracellular matrix. The typical materials found in the matrix are highlighted in a side box and cover both those that are produced and those that accumulate. The top panel of images showcases biofilms in the natural and built environment, which cross many scales. The lower panel of images highlight the emergent properties that arise when individual cells are in proximity within the protected community.

It is now accepted that the hallmark of a biofilm is the embedding of microorganisms within a matrix of extracellular polymeric substances that is produced by the constituent cells and additionally accumulates material (e.g., metals, salts) from the surrounding environment. The biofilm matrix is comprised of polysaccharides, proteins (many of which can be fibrous or form other higher-order forms), extracellular DNA and RNA, lipids, and other biomolecules that collectively form a protective and adhesive layer around the community (Figure 1). The specific matrix materials vary greatly and depend on the resident microbes and environmental conditions. For instance, *Pseudomonas aeruginosa* can produce at least three unique biofilm exopolysaccharides, and matrix production in *Bacillus subtilis* varies both with the isolate being examined and environmental factors such as temperature.

## Why do microbes form biofilms?

Biofilms exhibit emergent properties that arise from the interactions between the microorganisms that make up the community, as well as between the microorganisms and their environment. These emergent properties provide benefits to the microbial community, beyond the benefit to any individual cell (Figure 1). Some of the best-studied emergent properties include:

1. **Adhesion and structure:** Biofilms form on a variety of surfaces, both biotic and abiotic, and can create stable and robust attachments making removal difficult and hence enhancing survival. Mature biofilms are 3D space-occupying structures with complex architectures. This causes spatial gradients and distinct microenvironments that can vary in nutrient availability, oxygen concentration, and pH.
2. **Division of labour:** The formation of different microenvironments can lead to the emergence of different subpopulations of genetically identical microorganisms within the biofilm which each exhibit different behaviours and metabolic activities. Thus, the community can split tasks for maximum efficiency and cooperativity. However, inherent in this process is a need to protect the community from non-producing cells that, for example, can escape the metabolic burden of making shared goods but benefit from their production leading to population collapse.
3. **Nutrient cycling:** Within the matrix, biofilms can cycle and recycle nutrients, which can support the growth and survival of the microorganisms in the community. This nutrient cycling is facilitated by enzymes that are produced by the microorganisms in the biofilm, as well as physical and chemical interactions between the cells and the matrix. The process of nutrient cycling can occur both within a single isogenic community or between species.
4. **Enhanced robustness:** Residents within biofilms are often more tolerant to environmental perturbations than planktonic (free-living) microorganisms, due to the protective properties of the biofilm matrix and the ability of the microorganisms to adapt to changing environmental conditions. Microbes in biofilm communities often demonstrate decreased growth and increased antibiotic tolerance, pH, temperature and stress resistance, and immune evasiveness.
5. **Exchange of genetic material:** Biofilms are known hotspots for horizontal gene transfer, which allows the exchange of genetic material between different microorganisms of the same or different species. This exchange can lead to the rapid acquisition of new traits that allow the biofilm community to adapt to changing environmental conditions, including the enhanced development of antimicrobial resistance.
6. **Collective action:** Many microorganisms in biofilms use chemical signalling (called quorum sensing) to communicate with each other and coordinate their activities. This allows the microorganisms to regulate their behaviour in response to changes in the biofilm environment. For example, quorum sensing enables microbes to sense local cell density so that collective functions are only switched on or off when an appropriate number of cells are present to take advantage.

## Notable biofilms

Biofilms are highly adaptable and have been found in a wide range of extreme environments. We have collated examples of biofilms and highlighted the diverse impacts that they have on our natural and built environments and on human health (Figure 1).

### Biggest (and oldest) biofilms

The largest known biofilms are in the form of stratified microbial mats. The Great Sippewissett Salt Marsh in Massachusetts, USA hosts a microbial mat that is estimated to cover an area of over 1,000 square meters and can be several centimetres thick, depending on the season. On an even larger scale, the Great Salt Lake microbial mats in Utah, USA span over 700 square kilometres of a hypersaline environment. The mats are composed of many different types of microorganisms, including bacteria, archaea, and algae, which work together to form a complex ecosystem. Such mats can trap, bind, or precipitate sediment because of the growth and metabolic activity of the constituent organisms, resulting in lithification (rock formation, or fossilisation). The oldest known examples of such living fossils are the “lake stromatolites” in Western Australia, which are composed of ancient microbial mats that have been growing for over 3.6 billion years, emerging shortly after the Earth’s environment became compatible with life. The stromatolites cover an area of over 10 square kilometres and have a height of up to several meters.

### Dark cave biofilms

Dark cave biofilms, such as those in the Fornelle cave system in Italy, are often composed of a diverse community of microorganisms, including bacteria, archaea, fungi, and algae, and they can exhibit unique properties and adaptations that allow them to thrive in these extreme environments. Cave biofilms are often able to survive in nutrient-poor environments by utilizing alternative sources of energy such as inorganic compounds, like ammonia or sulphur, in a process known as chemolithotrophy. Cave biofilms can also contribute to the formation of mineral deposits, such as stalactites and stalagmites, through the production of the biofilm matrix that helps to bind and cement mineral particles together. Microbes dwelling in dark cave biofilms are being investigated as novel sources of antimicrobials.

### Most colourful biofilms

Yellowstone National Park, USA is home to a diverse range of colourful biofilms that inhabit the park’s thermal features, such as hot springs, geysers, and fumaroles. These biofilms are composed of a variety of microorganisms which have adapted to thrive in the extreme temperatures and chemical conditions found in these environments. The dramatic colours of the biofilms are due to the presence of pigments produced by the microorganisms, which can range from shades of orange and yellow to deep green and brown. Other types of biofilms found in Yellowstone include “green slime” mats, which are composed of photosynthetic bacteria and algae, and “white streamers” formed by the extremophilic *Hydrogenobaculum spp*., which can use hydrogen gas, hydrogen sulphide, and thiosulfate as energy sources and carbon dioxide as its sole carbon source. These biofilms play important ecological roles in the park’s thermal features, serving as the base of the food chain for other microorganisms and small invertebrates.

### Biofilms as food for migratory birds

During stopovers between destinations, migratory birds such as sandpipers, plovers, and other shorebirds are known to feed on biofilms in a variety of environments including coastal areas, wetlands, and other aquatic habitats. These biofilms are often rich in diatoms and fatty acids which help the birds build up their energy reserves. This rich source of nutrients is especially important during the birds’ long migrations in Spring and Autumn when other food sources may be scarce. The birds are well adapted to feeding on the nutrient-rich mudflats and tidal pools: sandpipers and dunlin, for example, have evolved bristled tongues which aid them in grazing on the paste-like coastal biofilms. One study has shown that about half of the western sandpipers’ energy budget is from biofilm consumption.

### Biofilms in glacial environments

Biofilms can have significant impacts on glacial areas, both in terms of their physical structure and ecological functions and are known to be major players in nutrient cycling and other key processes. The communities are known to play important roles in the cycling of carbon, nitrogen, and phosphorus, and can help to mobilize and make available these key nutrients to other organisms in the ecosystem. In some cases, biofilms may serve as the primary food source for higher organisms, such as invertebrates and small fish.

Biofilms formed on glaciers can also alter the surface properties of the ice. Biofilms can create dark pigments that absorb more sunlight and heat, leading to localized melting and changes in the albedo of the ice surface. This can lead to a feedback loop where the melting of the ice creates more favourable conditions for biofilm growth, which in turn accelerates melting.

### Biofilms on infrastructure

Microbes are adept at forming biofilms on abiotic surfaces including those present in large-scale infrastructure. Biofilms can form in water cooling systems, such as those in power plants and industrial facilities, and can cause a range of problems. Once established, biofilms can provide a reservoir for microbial growth and can reduce the efficiency of heat transfer in the cooling system, leading to increased energy consumption and decreased system performance. The growth of biofilm communities on the surfaces of metal pipework or barriers can also significantly alter local physicochemical conditions. These changes can include local alterations in parameters such as pH, salt concentration, oxygen concentration, organic compound concentration, redox potential, and solution conductivity. Such changes can lead to accelerated corrosion and increased risk of equipment failure in industrial settings. An extreme example of potentially problematic biofilm formation in large-scale infrastructure is the biofilms that form in nuclear reactor cooling systems, despite locally high radiation levels, and this can impact the performance and safety of the reactor.

### Biofilms in space

Biofilms have been found to form on various surfaces and materials in the International Space Station, showing that microorganisms can attach to surfaces and form communities in microgravity and in the absence of gravity-driven fluid flows. Fungi are known to thrive in the low-humidity environment of the space station, and bacteria can form biofilms in areas with moisture or organic matter. Biofilms of bacteria, including *Bacillus* and *Staphylococcus* species, have been found on surfaces including air filters, exercise equipment, and computer keyboards, and fungal biofilms have been found on air vents, water dispensers, and personal items of the crew.

## Notable impacts of biofilms

### Biofouling in marine environments

Biofouling on ship hulls is caused by the attachment and growth of marine organisms such as algae, bacteria, barnacles, mussels, and other organisms on the surface of the ship’s hull. The process of biofouling begins when microorganisms such as bacteria and diatoms attach themselves to the surface of the ship’s hull. As the organisms grow and reproduce, the biofilm matrix attaches the cells firmly to the hull. This leads to a slimy biofilm-coated surface that facilitates attachment of larger organisms.

The type and extent of biofouling can depend on several factors such as the location, nutrient levels, water temperature, salinity, currents, and the length of time a ship remains stationary in the water. Overall, biofouling is more common in warm waters where the growth rate of marine organisms is high.

Biofouling can have several negative impacts on ships, such as increased fuel consumption, reduced speed, and increased maintenance costs. It can also lead to the introduction of invasive species to new environments when ships travel from one area to another, which can have significant ecological and economic impacts. As a result, ship owners and operators take a variety of measures to prevent or mitigate biofouling, such as using antifouling coatings, cleaning the hull regularly, and implementing other best management practices. Biofilm prevention is a current focus as larger-scale biofouling depends on this first step.

### Dental plaque as the cause of caries and gum disease

Dental plaque is a type of biofilm that forms on the teeth and gums. It is a major cause of tooth decay and gum disease, which are two of the most common oral health problems worldwide. Dental plaque begins to form shortly after tooth surfaces are cleaned and is initially composed of a layer of salivary proteins and glycoproteins that adhere to the tooth surface. This layer provides an attachment site for bacteria, which begin to colonize and form a complex community of microorganisms which adhere in a predictable order. Collectively, the microorganisms in dental plaque can produce a variety of acids and enzymes that can damage the tooth enamel and contribute to the development of cavities. They can also trigger an inflammatory response in the gums, leading to gingivitis and periodontitis. The biogeography of the microbial communities on the tooth surface can have a significant impact on the outcome on the host.

### Biofilms as a source of chronic infections

Biofilms are frequently associated with chronic infections because they are difficult to eradicate with antimicrobial treatments and can evade the host’s immune system. In the case of chronic infections, such as those caused by *Pseudomonas aeruginosa, Staphylococcus aureus*, and *Escherichia coli*, biofilms can form on the surface of indwelling medical devices, such as catheters, implants, and prosthetics. These biofilms can act as a reservoir of bacteria that continuously release low levels of infectious agents, leading to persistent or recurring acute infections.

Biofilms can also contribute to chronic infections by promoting antibiotic tolerance among the residents. The extracellular matrix of the biofilm can provide a physical barrier that limits the diffusion of some antibiotics and other antimicrobial agents, making it difficult for them to reach the bacterial cells within the biofilm. Additionally, bacteria within the biofilm can adapt to the presence of antibiotics by activating genes whose products confer resistance to these drugs. At a more fundamental level, when residing within a biofilm microbes adopt a profile of gene regulation that is significantly different from their planktonic counterparts and one which triggers a multitude of adaptive responses that are associated with increased antimicrobial tolerance.

When formed, biofilms can trigger chronic inflammatory responses in the host, leading to tissue damage and impaired healing. The biofilm matrix can stimulate host immune cells to release inflammatory mediators, which can cause chronic inflammation and tissue damage.

### Biofilms in the Rhizosphere

Microbes including bacteria and mycorrhizal fungi form biofilms around the roots of plants (called the rhizosphere) and create a mutualistic relationship. Biofilms in the soil can help to break down organic matter and release nutrients such as nitrogen, phosphorus, and potassium, which are essential for plant growth. Biofilms can also improve soil structure and water retention, making it easier for plants to access water and nutrients. In return, the host plant provides the microbes with carbohydrates that they produce through photosynthesis.

A specialised example is rhizobial bacteria that form biofilms around the roots of leguminous plants, such as beans and peas and trigger the development of root nodules. From within the nodules, the bacteria fix nitrogen from the atmosphere, making it available to the plant. Plant exudates (secreted molecules) can act as chemoattractants for bacteria such as *Bacillus subtilis* leading to active root colonisation. Some plant exudates can induce biofilm formation by triggering chemoreceptors that start a signal cascade leading to biofilm matrix gene expression, demonstrating the complexity of the cross-Kingdom relationship. Current research is looking into how to promote beneficial biofilm formation on crop plants to increase plant robustness and crop yield, and how to breed crops that have the maximum potential of attracting beneficial microbes.

### Biofilms and bioremediation

Biofilms are diverse in their biological function and this diversity is currently being explored for the detoxification or treatment of human waste products. This process falls under bioremediation where biofilms can be harnessed to treat wastewater, oil spills, heavy metal contamination, pesticide removal, and even microplastics.

Activated sludge has been used to treat wastewater since the early 1900s. Over the last hundred years, scientists have determined that activated sludge is in fact a multispecies microbial biofilm. The microbes remove nitrogen and phosphorus from the wastewater, thus detoxifying it. Similarly, biofilms can remove heavy metals from contaminated soil caused by mining, industry, and other human activities. Not only can the biofilm matrix bind and absorb heavy metal ions, but there are also metal-tolerant species that uptake ions and convert them to a less toxic form. For instance, *Acinetobacter radioresistens* can reduce toxic chromate, Cr(VI), to a less toxic form, Cr(III). Bacterial biofilms have also been shown to absorb and metabolise pesticides used in agriculture.

Recently, research on microbial biofilms that adhere to and degrade microplastics has been expanding. In aquatic environments, microplastics are colonised by many types of microbes including bacteria, fungi, and algae, and some of these microbes produce enzymes that modify and hydrolyse plastic polymers. Some species, like *Comanonas*, have been further shown to uptake the degradation products through active transporters. The exploitation of biofilms for the removal of microplastics from the environment is a critical area of research.

## Prospects

Our need to engineer and manage biofilms in complex built and natural environments will remain a priority in the coming decades. There is the potential for biofilm innovations to provide solutions to many of our most pressing problems that cross healthcare to sustainability challenges. Foundational discovery-based research will drive solutions, but we will need to be creative as microbes have the collective advantage of millions of years of evolution and cooperation to draw on and resist or perturb any interventions.
